# Risk of subclinical atherosclerosis across metabolic transition in individuals with or without fatty liver disease: a prospective cohort study

**DOI:** 10.1186/s12986-023-00734-3

**Published:** 2023-03-10

**Authors:** Zhuojun Xin, Jiaojiao Huang, Qiuyu Cao, Jialu Wang, Ruixin He, Tianzhichao Hou, Yi Ding, Jieli Lu, Tiange Wang, Zhiyun Zhao, Weiqing Wang, Guang Ning, Min Xu, Yufang Bi, Yu Xu, Mian Li

**Affiliations:** 1 Department of Endocrine and Metabolic Diseases, Shanghai Institute of Endocrine and Metabolic Diseases, Ruijin Hospital, Shanghai Jiao Tong University School of Medicine, Shanghai, China; 2 Shanghai National Clinical Research Center for Metabolic Diseases, Key Laboratory for Endocrine and Metabolic Diseases of the National Health Commission of the People’s Republic of China, Shanghai Key Laboratory for Endocrine TumorState Key Laboratory of Medical GenomicsShanghai National Center for Translational Medicine, Ruijin Hospital, Shanghai Jiao Tong University School of Medicine, 197 Ruijin 2Nd Road, Shanghai, 200025 China

**Keywords:** Fatty liver disease, Metabolic dysfunction, Metabolic transition, Subclinical atherosclerosis

## Abstract

**Background:**

Metabolic dysfunction is a major determinant in the progression of fatty liver disease. It is pivotal to evaluate the metabolic status and subsequent transition in fatty liver population and to identify the risk of subclinical atherosclerosis.

**Methods:**

The prospective cohort study included 6260 Chinese community residents during 2010–2015. Fatty liver was determined as hepatic steatosis (HS) by ultrasonography. Metabolic unhealthy (MU) status was defined as having diabetes and/or ≥ 2 metabolic risk factors. Participants were categorized into 4 groups according to the combination of metabolic healthy (MH)/MU and fatty liver status (MHNHS, MUNHS, MHHS and MUHS). Subclinical atherosclerosis was assessed by elevated brachial-ankle pulse wave velocity, pulse pressure and/or albuminuria.

**Results:**

31.3% of the participants had fatty liver disease and 76.9% were in MU status. During a 4.3-year follow-up, 24.2% of participants developed composite subclinical atherosclerosis. Multivariable adjusted odds ratios for composite subclinical atherosclerosis risk were (1.66 [1.30–2.13]) in MUNHS group and (2.57 [1.90–3.48]) in MUHS group. It seemed that participants with fatty liver disease were more prone to be remained in MU status (90.7% *vs.*50.8%) and less likely to regress to MH status (4.0% *vs.* 8.9%). Fatty liver participants progressed to (3.11 [1.23–7.92]) or maintained MU status (4.87 [3.25–7.31]) significantly impelled the development of the composite risk, while regressing to MH status (0.15 [0.04–0.64]) were more intended to mitigate the risk.

**Conclusions:**

The current study emphasized the importance of assessing metabolic status and its dynamic changes, especially in the fatty liver population. Regressing from MU to MH status not only benefited the systematic metabolic profile but also ameliorated future cardiometabolic complications.

**Supplementary Information:**

The online version contains supplementary material available at 10.1186/s12986-023-00734-3.

## Introduction

Globally, fatty liver disease has become an epidemic, affecting approximately 25% of adult population [1]. Prevailing fatty liver disease poses individuals at increased risk of both end-stage liver diseases and extra-hepatic complications [2–5]. Accumulating evidence has indicated the critical role of metabolic dysregulation in the process of adverse prognosis, for which proposed the new terminology of metabolic dysfunction-associated fatty liver disease (MAFLD) [6].

There is a substantial overlap between non-alcoholic fatty liver disease (NAFLD) and MAFLD [7, 8]. Considering the strong association with body mass index (BMI), fatty liver patients characterized overweight or obesity are included into MAFLD criteria, which accounted for more than 50% [8, 9]. Recently, a cross-sectional cohort study further pointed out that a considerable proportion (26.6%) of this subgroup were metabolic healthy, referring to the absence of type 2 diabetes and metabolic risk factors of MAFLD criteria [10]. In view of its dynamic feature, the presence of metabolic healthy status emphasized the importance of holistically assessing the metabolic status and further metabolic transition among fatty liver population.

Mounting data supported the strong link of fatty liver disease with atherosclerotic cardiovascular disease as well as subclinical markers of atherosclerosis [11–13]. It has been suggested that fatty liver patients at a high risk of cardiovascular progression is more frequently associated with metabolic abnormality. Moreover, fatty liver disease is not only manifested by excessive liver fat deposition, but also accompanied with metabolic abnormalities, leading to a highly heterogeneous condition [14]. The concept of MAFLD also emphasized the importance of metabolic heterogeneity. However, there is few data covering the metabolic transition towards fatty liver population, and it remains unclear to what extent that fatty liver population with varying phenotypes of metabolic status and metabolic transition are associated with the risk of subclinical atherosclerosis, an early lesion status of cardiovascular disease, such as arterial stiffness, coronary calcification and endothelial dysfunction.

The aim of this study was firstly to confirm the distribution of metabolic healthy/metabolic unhealthy (MH/MU) status among participants with ultrasound-based hepatic steatosis, and to evaluate the individual and combined associations of fatty liver disease and MH/MU status as well as metabolic transition with the risk of incident subclinical atherosclerosis, and to further discuss the potential effect and related determinants of MU regression on the established prognosis.

## Patients and methods

### Study design and population

**Fig. 1:**
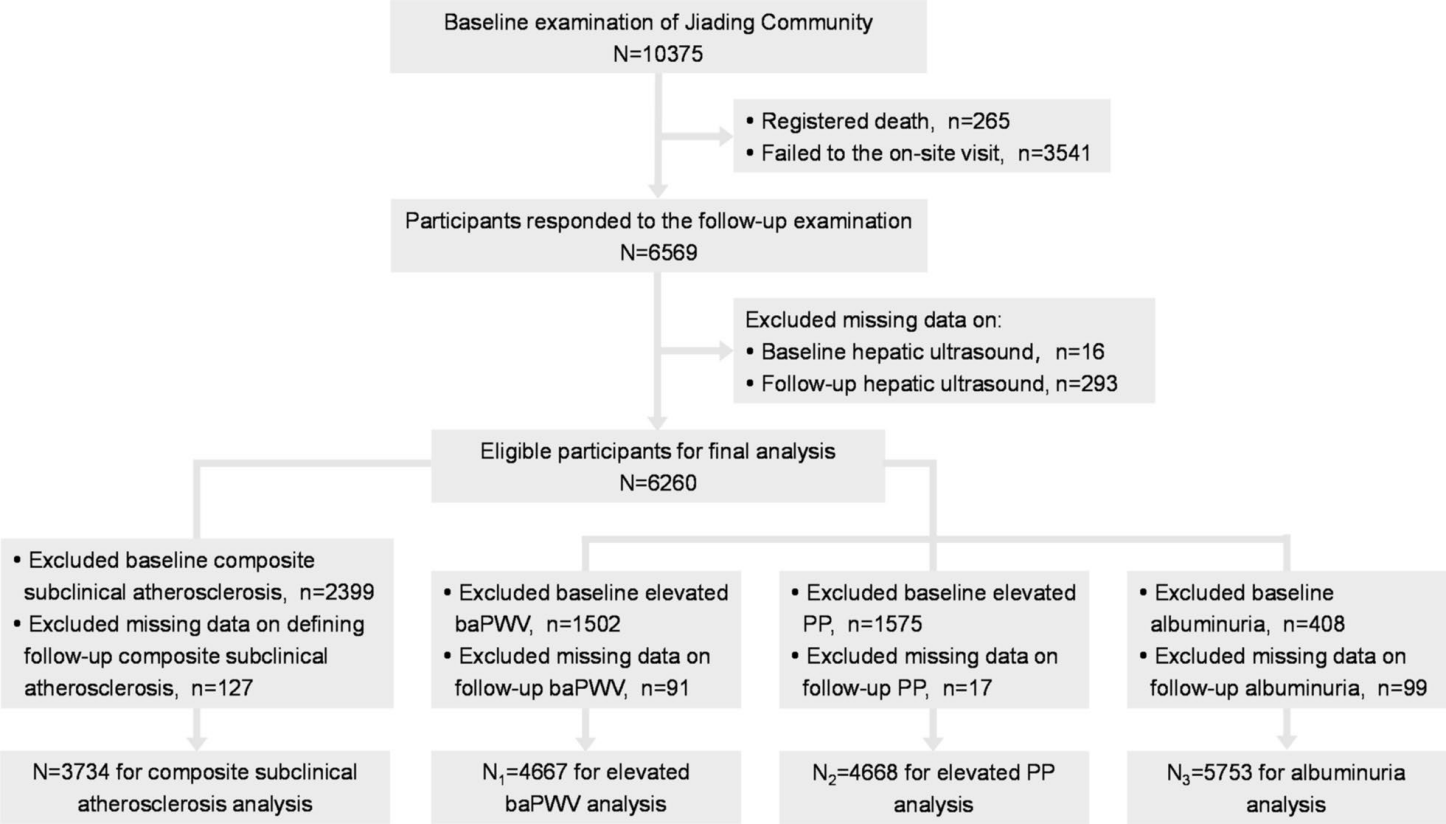
Study population flow diagram. BaPWV, brachial–ankle pulse wave velocity; PP, pressure pulse

The study population was from a community-based cohort study in Jiading District of Shanghai, China. The detailed protocol has been published previously [15]. In brief, the cohort study was launched among 10,375 permanent residents (≥ 40 years old) between March and August 2010. Baseline health examinations comprising of a standard questionnaire and clinical measurements were completed for each participant. After a follow-up interval for up to 5 years, participants were re-invited for an on-site visit during August 2014 and May 2015. For the current study, we excluded individuals who registered for death at follow-up (n = 265), failed to the on-site follow-up visit (n = 3541) and had missing data on the baseline (n = 16) or follow-up (n = 293) hepatic ultrasound, leaving 6260 for ensuing analysis. In the separate outcome analysis, pre-existing subclinical atherosclerosis at baseline and data missing at follow-up reflected by elevated brachial-ankle pulse wave velocity (baPWV) (n = 1593), elevated pulse pressure (PP) (n = 1592) and albuminuria (n = 507) were further excluded from baPWV, PP and albuminuria analyses, respectively. 2526 were additionally excluded with any of elevated baPWV, elevated PP or albuminuria or respective missing data from the composite subclinical atherosclerosis analysis. Detailed selection flowchart was presented in Fig. 1. The study protocol conformed to the ethical guidelines of the 1975 Declaration of Helsinki and was approved by the Institutional Review Board of Ruijin Hospital. Enrolled participants signed written informed consents.

### Data collection

Standard questionnaires were administered to collect information regarding individuals’ demographic characteristics, medical history, cigarette smoking, alcohol intake and physical activity both at baseline and follow-up interview. Physical activity was assessed by the short form of the Global Physical Activity Questionnaire and calculated into metabolic equivalent minutes per week (MET-min/wk) [16]. The anthropometry data including body weight, height and waist circumference were measured by trained staff, with a standard protocol applied. Automated electronic blood pressure monitoring was used with Omron model HEM-752 FUZZY. The average of three seated measurements was adopted for analysis. PP was defined as systolic blood pressure (SBP) minus diastolic blood pressure (DBP) from the average of the three readings. BMI was calculated as body weight (kg) divided by square of the height (m^2^).

Blood samples were drawn after ≥ 10 h of fasting to evaluate levels of fasting plasma glucose, triglycerides, total cholesterol, low-density lipoprotein cholesterol (LDL-C), high-density lipoprotein cholesterol (HDL-C) and liver enzymes (including aspartate transaminase, alanine transaminase, and gamma-glutamyl transferase). Then, participants underwent a standard 75-g load oral glucose tolerance test to examine 2-h postprandial plasma glucose by the glucose oxidase method on an automated analyzer (Modular Analytics P800; Roche). Glycated hemoglobin (HbA1c) was determined by high-performance liquid chromatography using the VARIANT II Hemoglobin Testing System (Bio-Rad Laboratories). Plasma lipids and liver enzymes were assessed by an automated analyzer (Modular E170; Roche). The index of homeostasis model assessment of insulin resistance (HOMA-IR) was calculated as fasting serum insulin (μIU/mL) × fasting plasma glucose (mmol/L)/22.5. The cutoff of HOMA-IR ≥ 2.5 was defined as insulin resistance [17].

A first-voided, early-morning spot urine sample was obtained for the measurement of urinary albumin (mg/dL) and creatinine (mmol/L) and tested by the immunoturbidimetric method (Beijing Atom High-Tech, Beijing, China) and Jaffe’s kinetic method on an automatic analyzer (Hitachi 7600–020, Tokyo, Japan), respectively. Women experiencing menstruation on the survey day were not included in the present study. Urinary albumin-to-creatinine ratio (UACR) was calculated by dividing the urinary albumin concentrations by the urinary creatinine concentrations and presented in mg/g.

All participants underwent baPWV measurement at baseline and follow-up visit on Colin VP-1000 (Model BP203RPE II, form PWV/ABI) after 10-min rest for the evaluation of artery stiffness. Pulse waves were obtained simultaneously with suitable cuffs placed on the upper sides of bilateral arms and ankles. The distance from bilateral upper arms to ankles was corrected for its difference of time delay when obtaining the baPWV. The greater value of bilateral baPWV was adopted for analysis.

### Assessment of MU status and fatty liver disease

Criteria of metabolic risk factors were defined according to MAFLD consensus as the following [6]: (1) waist circumference ≥ 90/80 cm; (2) blood pressure ≥ 130/85 mmHg and/or taking antihypertensive medication; (3) triglycerides ≥ 150 mg/dl and/or taking lipid-lowering medication; (4) HDL-C < 40 mg/dl for men and HDL-C < 50 mg/dl for women or taking lipid-lowering medication; (5) prediabetes: fasting plasma glucose (FPG) 100 to 125 mg/dl or 2 h-postprandial glucose (2 h-PG) 140 to 199 mg/dl, or HbA1c 5.7% to 6.4%; and (6) HOMA-IR ≥ 2.5. MU status was defined as having type 2 diabetes (FPG ≥ 126 mg/dl or 2 h-PG ≥ 200 mg/dl, or HbA1c ≥ 6.5% or taking hypoglycemic medicine) and/or ≥ 2 metabolic risk factors [10].

Liver ultrasound was operated by two radiologists who were blinded to the protocol, using a high-resolution B-mode tomographic ultrasonic system (Esaote Biomedica SpA, Italy) with a 3.5-MHz probe. Fatty liver disease was determined as hepatic steatosis by the presence of ≥ 2 of 3 abnormal imaging findings: diffusely increased echogenicity (‘bright’) liver—with liver echogenicity greater than kidney or spleen, vascular blurring, and deep attenuation of ultrasound signal [18].

Study participants were divided into four groups according to baseline MH/MU and fatty liver status: (1) metabolic healthy and no hepatic steatosis, MHNHS; (2) metabolic unhealthy and no hepatic steatosis, MUNHS; (3) metabolic healthy and hepatic steatosis, MHHS; and (4) metabolic unhealthy and hepatic steatosis, MUHS.

### Assessment of subclinical atherosclerosis

Subclinical atherosclerosis was separately defined by elevated baPWV, elevated PP and albuminuria [19–22]. The combination pattern was regarded as the composite outcome. Baseline and incident elevated baPWV and elevated PP were referred to upper quartiles of baseline baPWV (≥ 1768.0 cm/s) and baseline PP (≥ 67.3 mmHg). Incident albuminuria was defined as UACR ≥ 30 mg/g.

### Statistical analysis

For statistic description, means ± standard deviations or medians (interquartile ranges) were fitted for continuous variables and numbers (proportions) for categorical variables. HOMA-IR, triglycerides, alanine aminotransferase, aspartate transaminase, gamma-glutamyl transferase and UACR were logarithmically transformed to achieve a normal distribution. Differences of baseline characteristics among the four groups were determined using one-way ANOVA and chi-square test.

We adopted multivariable logistic regression analysis to assess the longitudinal associations of baseline metabolic health and fatty liver status as well as metabolic status changes with incident subclinical atherosclerosis (separate/composite). We selected a priori potential confounders for adjustment in multivariable models based on knowledge of their associations with MAFLD and subclinical atherosclerosis. Potential confounders including age, sex, follow-up interval (Model 1), current smoking and drinking status (yes/no), education (≥ 12 years or not), log-transformed physical activity, baseline BMI and BMI change (Model 2), were adjusted in the analysis models.

To further illustrate the metabolic regression of MUHS group during follow-up, changes of metabolic risk parameters between baseline and follow-up visits were compared between the stable MU and MU to MH groups. Logistic regression models with generalized estimating equations were used to explore the associated metabolic parameters in metabolic regression. For repeated-measures analysis, the multivariable model was adjusted for age, sex, current smoking and drinking status (yes/no), education (≥ 12 years or not), log-transformed physical activity, waist circumference, SBP, DBP, triglycerides, HDL-C, FPG, 2 h-PG, HbA1c, HOMA-IR and BMI. All covariates except sex and education were repeated-measured both at baseline and follow-up and modelled as time-varying variables.

Sensitivity analysis was conducted (1) in exclusion of participants receiving treatment with hypoglycemic medications or insulin, blood pressure- or lipid-lowering medications during the baseline and follow-up, eliminating the interference of metabolic related medications on the metabolic transition; (2) in exclusion of participants with advanced stage assessed by Fibrosis-4 score > 2.67, excessive alcohol consumption and other liver diseases, specifying the actual relationship of simple fatty liver disease combining with metabolic abnormality with risk of subclinical atherosclerosis.

Results were represented as odds ratios (OR) with 95% confidence intervals (CI), with a 2-tailed alpha value of 0.05 considered statistically significant. Statistical analysis was performed on SAS 9.2 (SAS Institute, Cary, NC).

## Results

### Baseline characteristics of study population

Overall, 31.3% of the participants had ultrasound-detected hepatic steatosis and 76.9% were categorized as MU status. Participants with hepatic steatosis but remaining in MH status (MHHS group) accounted for 1.0% among the baseline population (n = 6260). Compared with MHNHS group, MHHS group displayed a slightly unfavorable metabolic profile, including higher levels of BMI, waist circumference, blood pressure, plasma glucose, lipid and liver enzymes, but younger age, higher educational attainment and more time occupied in physical activity. The aforementioned metabolic profile along with baPWV, PP and UACR were significantly worse in MU groups, irrespective of fatty liver status. Additionally, participants with fatty liver performed higher levels of BMI and waist circumference, as well as liver enzymes than no fatty liver counterparts (all *P* < 0.05). Baseline sociodemographic and biochemical characteristics of the entire study population were presented in Table 1.

**Table 1:** Baseline characteristics of the study population according to MH/MU and fatty liver status (N = 6260)

	No fatty liver (4301, 68.7%)		Fatty liver (1959, 31.3%)		*P* value
	MHNHS	MUNHS	MHHS	MUHS	
No. of participants, n (%)	1382 (22.1)	2919 (46.6)	63 (1.0)	1896 (30.3)	< 0.0001
Age (years)	54.7 ± 8.4	59.0 ± 8.7	53.6 ± 6.5	58.0 ± 8.2	< 0.0001
Male sex, n (%)	545 (39.4)	995 (34.1)	36 (57.1)	688 (36.3)	< 0.0001
High school and above, n (%)	353 (25.5)	511 (17.5)	19 (30.2)	374 (19.7)	< 0.0001
Current drinking, n (%)	310 (22.4)	516 (17.7)	22 (34.9)	366 (19.3)	< 0.0001
Current smoking, n (%)	366 (26.5)	596 (20.4)	27 (42.9)	422 (22.3)	< 0.0001
Vigorous activity ≥ 75 min/week or moderate-vigorous ≥ 150 min/week, n (%)	203 (14.7)	457 (15.7)	11 (17.5)	343 (18.1)	0.0454
Hypertension, n (%)	353 (25.5)	1921 (65.8)	15 (23.8)	1407 (74.2)	< 0.0001
Anti-hypertensive medications, n (%)	118 (8.6)	876 (30.0)	5 (8.1)	790 (41.6)	< 0.0001
Type 2 diabetes, n (%)	0 (0)	516 (17.7)	0 (0)	691 (36.5)	< 0.0001
Anti-diabetic medications or insulin, n (%)	0 (0)	234 (8.0)	0 (0)	224 (11.8)	< 0.0001
Dyslipidemia, n (%)	210 (15.2)	1239 (42.5)	17 (27.0)	1179 (62.2)	< 0.0001
Lipid lowering medications, n (%)	0 (0)	7 (0.2)	0 (0)	7 (0.4)	0.1665
Body mass index (kg/m^2^)	22.8 ± 2.3	24.9 ± 2.8	25.3 ± 2.0	27.6 ± 3.0	< 0.0001
Waist circumference (cm)	75.6 ± 6.5	81.9 ± 7.5	82.8 ± 5.7	89.2 ± 7.7	< 0.0001
SBP (mmHg)	127.7 ± 17.2	144.2 ± 18.5	128.6 ± 15.9	147.1 ± 19.0	< 0.0001
DBP (mmHg)	77.6 ± 9.4	83.9 ± 9.8	80.0 ± 8.6	86.1 ± 10.1	< 0.0001
PP (mmHg)	50.1 ± 12.8	60.4 ± 15.4	48.6 ± 10.3	61.0 ± 16.1	< 0.0001
Fasting plasma glucose (mg/dL)	87.8 ± 8.0	99.4 ± 24.0	89.3 ± 9.0	110.4 ± 35.5	< 0.0001
Postprandial plasma glucose (mg/dL)	103.9 ± 23.8	146.4 ± 72.7	110.6 ± 28.5	185.3 ± 91.8	< 0.0001
HbA1c (%)	5.4 ± 0.3	5.8 ± 0.8	5.5 ± 0.3	6.2 ± 1.2	< 0.0001
HOMA-IR	1.0 (0.7–1.4)	1.5 (1.1–2.2)	1.4 (1.0–1.9)	2.6 (1.8–3.8)	< 0.0001
Triglycerides (mg/dL)	86.7 (68.1–111.5)	123.9 (90.3–169.0)	105.3 (84.1–125.7)	167.3 (121.2–232.3)	< 0.0001
LDL-C (mg/dL)	114.0 ± 28.6	126.2 ± 33.6	123.3 ± 31.0	128.2 ± 35.3	< 0.0001
HDL-C (mg/dL)	57.9 ± 11.0	50.8 ± 12.3	54.6 ± 11.5	46.4 ± 10.4	< 0.0001
Total cholesterol (mg/dL)	196.8 ± 33.2	208.1 ± 38.7	204.8 ± 35.8	213.2 ± 42.2	< 0.0001
ALT (IU)	15.6 (12.3–20.8)	17.1 (13.4–22.5)	19.2 (15.0–25.9)	23.8 (17.6–34.7)	< 0.0001
AST (IU)	20.9 (18.0–24.8)	21.1 (18.2–24.7)	22.0 (17.3–25.5)	22.4 (19.1–27.6)	< 0.0001
GGT (IU)	16.0 (12.0–24.0)	20.0 (14.0–29.0)	26.0 (15.0–48.0)	30.0 (21.0–47.5)	< 0.0001
BaPWV (cm/s)	1419.6 ± 284.9	1634.8 ± 350.3	1426.5 ± 218.5	1678.1 ± 357.7	< 0.0001
UACR (mg/g)	4.0 (2.4–6.6)	4.8 (2.8–8.8)	3.6 (2.4–5.1)	5.9 (3.2–11.9)	< 0.0001

Values are means ± SD, medians (interquartile ranges) or numbers (proportions)

*SBP* systolic blood pressure; *DBP* diastolic blood pressure; *PP* pulse pressure; *HbA1c* glycated hemoglobin; *HOMA-IR* homeostasis model assessment of insulin resistance; *LDL-C* low density lipoprotein cholesterol; *HDL-C* high density lipoprotein cholesterol; *ALT* alanine aminotransferase; *AST* aspartate aminotransferase; *GGT* gamma-glutamyl transferase; *baPWV* brachial–ankle pulse wave velocity; *UACR* urinary albumin-to-creatinine ratio; *MHNHS* metabolic healthy and no hepatic steatosis; *MUNHS* metabolic unhealthy and no hepatic steatosis; *MHHS* metabolic healthy and hepatic steatosis; *MUHS* metabolic unhealthy and hepatic steatosis

*P* values were calculated from one-way ANOVA for continuous variables and χ^2^ test for categorical variables

### Risk of incident subclinical atherosclerosis (composite/separate) for participants with fatty liver in relation to the number of metabolic abnormalities

**Fig. 2:**
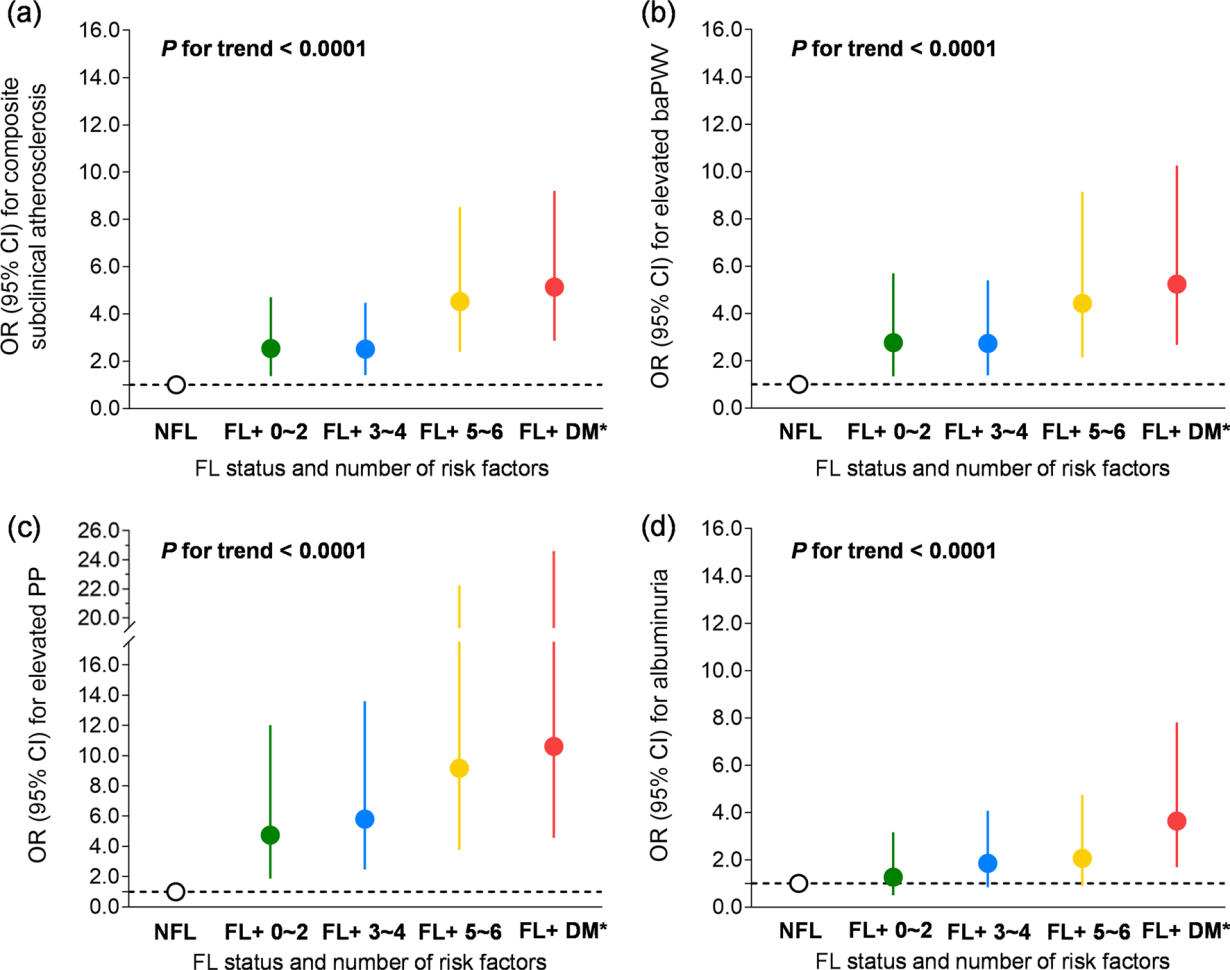
Risk of incident subclinical atherosclerosis (composite/separate) for participants with fatty liver in relation to the number of metabolic abnormalities ORs (95% CIs) were adjusted for age, sex, follow-up interval, current smoking and drinking status (yes/no), education (≥ 12 years or not), log-transformed physical activity, baseline BMI and BMI change. *NFL* no fatty liver; *FL* fatty liver; *DM* diabetes mellitus; *baPWV* brachial–ankle pulse wave velocity; *PP* pressure pulse; *OR* odds ratio; *CI* confidential interval; *BMI* body mass index. *This group included type 2 diabetes participants with/without metabolic abnormalities

Figure 2 presented the associations of fatty liver combining increasing numbers of metabolic abnormalities (including metabolic risk factors and presence of diabetes) with incident subclinical atherosclerosis. Compared with participants without fatty liver or type 2 diabetes or any metabolic risk factors, fatty liver participants with increasing numbers of metabolic risk factors and diabetes tended to pose gradually incremental risk on the composite subclinical atherosclerosis (*P* for trend < 0.0001). The risk was most significant in the subgroup of combining with diabetes and metabolic risk factors (OR 5.14, 95% CI 2.87–9.20). Similar significance and tendency were simultaneously observed in the risk of elevated baPWV (OR 5.24, 95% CI 2.68–10.26) and elevated PP (OR 10.61, 95% CI 4.58–24.60), whereas it was only prominent in the combination subgroup of albuminuria analysis (OR 3.64, 95% CI 1.69–7.81). Those findings preliminarily indicated the dose-dependent effect of metabolic abnormalities on the poor prognosis of fatty liver population.

### Risk of subclinical atherosclerosis (composite/separate) according to combination of baseline MH/MU and fatty liver status

As shown in Table 2, during a median follow-up period of 4.3 years, 24.2% (902/3734) of composite subclinical atherosclerosis were recorded, including 17.7% (827/4667), 13.4% (627/4668) and 8.3% (476/5753) of elevated baPWV, elevated PP and albuminuria, respectively. Compared with MHNHS group, participants in MU status had an increased risk of incident composite subclinical atherosclerosis ([OR 1.66, 95% CI 1.30–2.13] for MUNHS group and [OR 2.57, 95% CI 1.90–3.48] for MUHS group) after full adjustments. Similar detrimental effects were investigated in the separate analysis of elevated baPWV and elevated PP. But for albuminuria, the risk was observed only in MUHS group (OR 1.81, 95% CI 1.23–2.65). Furthermore, there was no significant association observed in MHHS group with regards to the risk of composite or separate subclinical atherosclerosis. The results tended to be more significant when excluding advanced stage, excessive alcohol consumption and other liver diseases (Additional file 1: Table S1).

**Table 2:** Risk of incident subclinical atherosclerosis (composite/separate) according to MH/MU and fatty liver status at baseline

	N	Case, n (%)	Model 1 OR (95% CI)	*P* value	Model 2 OR (95% CI)	*P* value
*Composite Subclinical atherosclerosis (902/3734, 24.2%)*						
MHNHS	1092	152 (13.9)	1.00	–	1.00	–
MUNHS	1617	439 (27.2)	1.95 (1.58–2.41)	< 0.0001	1.66 (1.30–2.13)	< 0.0001
MHHS	54	10 (18.5)	1.57 (0.76–3.25)	0.2267	1.56 (0.68–3.56)	0.2907
MUHS	971	301 (31.0)	2.60 (2.07–3.27)	< 0.0001	2.57 (1.90–3.48)	< 0.0001
*Elevated baPWV (827/4667, 17.7%)*						
MHNHS	1231	118 (9.6)	1.00	–	1.00	–
MUNHS	2107	430 (20.4)	1.88 (1.50–2.37)	< 0.0001	1.82 (1.38–2.40)	< 0.0001
MHHS	57	6 (10.5)	1.29 (0.53–3.17)	0.5734	1.69 (0.62–4.57)	0.3043
MUHS	1272	273 (21.5)	2.34 (1.83–2.99)	< 0.0001	2.48 (1.78–3.45)	< 0.0001
*Elevated PP (627/4668, 13.4%)*						
MHNHS	1247	71 (5.7)	1.00	–	1.00	–
MUNHS	2078	338 (16.3)	2.55 (1.94–3.35)	< 0.0001	2.42 (1.75–3.36)	< 0.0001
MHHS	58	4 (6.9)	1.49 (0.52–4.31)	0.4594	2.08 (0.70–6.20)	0.1890
MUHS	1285	214 (16.7)	2.83 (2.12–3.77)	< 0.0001	2.92 (2.00–4.26)	< 0.0001
*Albuminuria (476/5753, 8.3%)*						
MHNHS	1302	70 (5.4)	1.00	–	1.00	–
MUNHS	2684	211 (7.9)	1.18 (0.88–1.57)	0.2684	1.09 (0.77–1.53)	0.6276
MHHS	63	3 (4.8)	1.00 (0.30–3.28)	0.9939	0.85 (0.20–3.63)	0.8233
MUHS	1704	192 (11.3)	2.01 (1.50–2.69)	< 0.0001	1.81 (1.23–2.65)	0.0024

Model 1 was adjusted for age, sex and follow-up interval;

Model 2 was further adjusted for current smoking and drinking status (yes/no), education (≥ 12 years or not), log-transformed physical activity, baseline BMI and BMI change

*MHNHS* metabolic healthy and no hepatic steatosis; *MUNHS* metabolic unhealthy and no hepatic steatosis; *MHHS* metabolic healthy and hepatic steatosis; *MUHS* metabolic unhealthy and hepatic steatosis; *baPWV* brachial–ankle pulse wave velocity; *PP* pulse pressure; *OR* odds ratio; *BMI* body mass index

### Transition of metabolic status and the risk of composite subclinical atherosclerosis

**Fig. 3:**
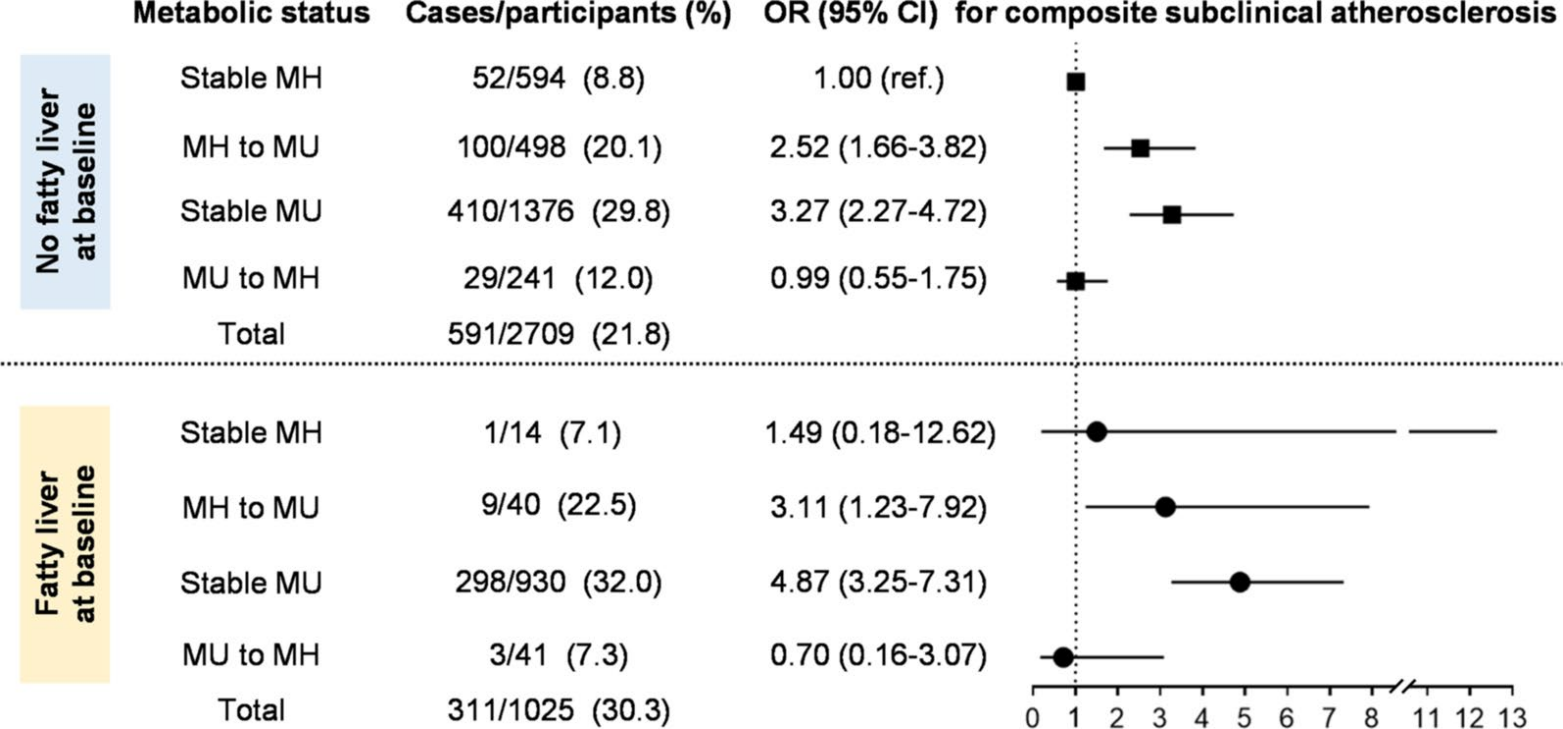
ORs (95% CIs) of composite subclinical atherosclerosis across varying transition of metabolic status. ORs (95% CIs) were adjusted for age, sex, follow-up interval, current smoking and drinking status (yes/no), education (≥ 12 years or not), log-transformed physical activity, baseline BMI and BMI change. *MH* metabolic healthy; MU, metabolic unhealthy; *OR* odds ratio; *CI* confidential interval; *BMI* body mass index

Considering the transient nature of MH status, the transitional trajectory was further investigated among the general population. Overall, 17.6% of the participants experienced metabolic transition and those who moved to or stayed in MU status tended to be older, with lower educational attainment and concomitantly higher levels of adipose, glucose, blood pressure and lipid parameters at baseline (Additional file 1: Table S2). When dividing the baseline population into two groups based on the presence of fatty liver at baseline, it seemed that participants with fatty liver disease were more prone to be remained in MU status (90.7% [930/1025] for fatty liver group *vs.*50.8% [1376/2709] for no fatty liver group) and less likely to regress to MH status (4.0% [41/1025] for fatty liver group *vs.* 8.9% [241/2709] for no fatty liver group) (Fig. 3).

Taken the participants without fatty liver at baseline and maintaining MH status for reference, progressing from MH to MU status ([OR 2.52, 95% CI 1.66–3.82] for no fatty liver group and [OR 3.11, 95% CI 1.23–7.92] for fatty liver group) and maintaining MU status ([OR 3.27, 95% CI 2.27–4.72] for no fatty liver group and [OR 4.87, 95% CI 3.25–7.31] for fatty liver group) contributed to an increased risk of composite subclinical atherosclerosis, irrespective of fatty liver status at baseline. Additionally, it was noteworthy that the composite risk was not significantly increased when regressing to MH status. Sensitivity analysis showed comparable results when excluding the influence of metabolic related medications as well as advanced stage and other etiologies of fatty liver disease (Additional file 1: Tables S3 and S4).

### Modified effect on the risk of subclinical atherosclerosis when regressing from MU to MH status and associated improvement of metabolic risk factors

**Fig. 4:**
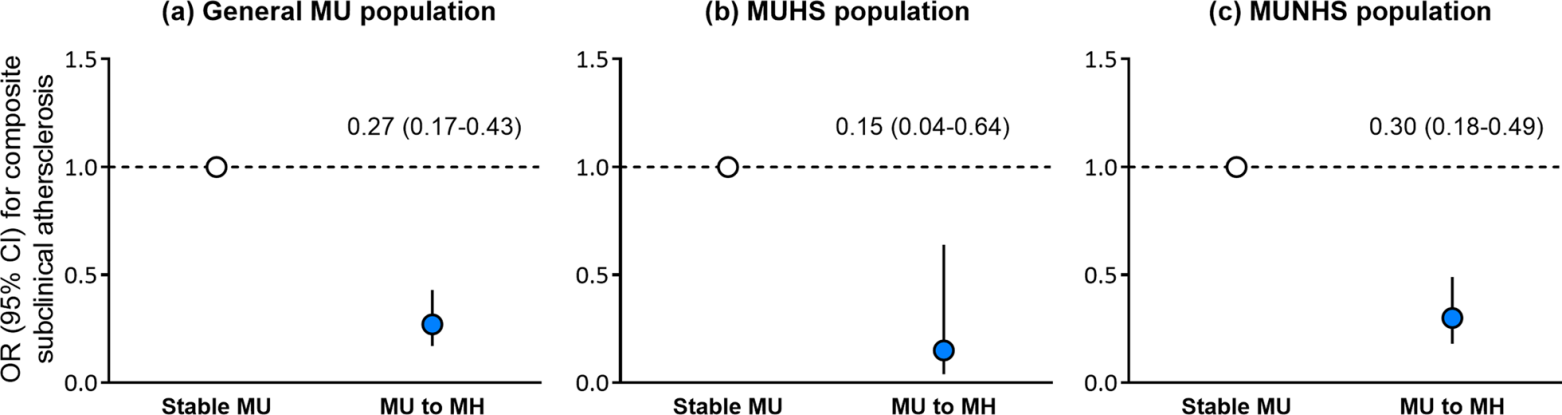
Modified effect of MU regression on the risk of composite subclinical atherosclerosis. ORs (95% CIs) were adjusted for age, sex, follow-up interval, current smoking and drinking status (yes/no), education (≥ 12 years or not), log-transformed physical activity, baseline BMI and BMI change. *MH* metabolic healthy; *MU* metabolic unhealthy; *MUHS* metabolic unhealthy and no hepatic steatosis; *MUNHS* metabolic unhealthy and hepatic steatosis; *OR* odds ratio; *CI* confidential interval; *BMI* body mass index

In the composite outcome analysis, there were 282 out of 2588 participants with MU status regressed to MH status, of which 32 (11.4%) developed the established outcome, 3/41 (7.3%) for MUHS group and 29/241 (12.0%) for MUNHS group, respectively. In general, MU regression modified the risk of composite subclinical atherosclerosis compared with those remaining in MU status (OR 0.27, 95% CI 0.17–0.43). This modification seemed to be more prominent in participants with fatty liver (OR 0.15, 95% CI 0.04–0.64) than non-counterparts (OR 0.30, 95% CI 0.18–0.49) and independent of metabolic medications as well as advanced stage and other etiologies of fatty liver disease (Fig. 4, Additional file 1: Tables S5 and S6). Separate analysis towards the risk of elevated baPWV, elevated PP and albuminuria were generally consistent with the composite outcome (Additional file 1: Tables S7 and S8).

Among baseline MUHS participants (n = 1896), 48 (2.5%) regressed to MH status. In comparison with stable MU group, participants regressing to MH status tended to display a more significant improvement in repeated-measured levels of waist circumference, SBP, triglycerides, HDL-C, HbA1c, fasting and postprandial plasma glucose after full adjustments (Table 3). Specific changes of metabolic risk factors were presented in Additional file 1: Table S9.

**Table 3:** Adjusted ORs (95% CI) of metabolic regression with repeated-measured metabolic risk factors among baseline MUHS group

	Age- and sex- adjusted		Multivariable-adjusted^†^	
	OR (95% CI)	*P* value	OR (95% CI)	*P* value
Waist circumference, cm	0.92 (0.90–0.95)	< 0.0001	0.91 (0.86–0.97)	0.0015
SBP, mmHg	0.95 (0.93–0.97)	< 0.0001	0.95 (0.92–0.98)	0.0012
DBP, mmHg	0.95 (0.92–0.97)	< 0.0001	0.998 (0.95–1.05)	0.9285
Triglycerides, mg/dL	0.49 (0.31–0.75)	0.0013	0.47 (0.23–0.96)	0.0393
HDL-C, mg/dL	6.08 (2.86–12.94)	< 0.0001	3.72 (1.13–12.28)	0.0307
FPG, mg/dL	0.42 (0.29–0.62)	< 0.0001	0.51 (0.33–0.79)	0.0022
2 h-PG, mg/dL	0.75 (0.71–0.78)	< 0.0001	0.79 (0.70–0.89)	< 0.0001
HbA1c, %	0.13 (0.07–0.23)	< 0.0001	0.21 (0.08–0.55)	0.0015
HOMA-IR	0.49 (0.38–0.64)	< 0.0001	0.77 (0.54–1.10)	0.1545
BMI, kg/m^2^	0.78 (0.70–0.87)	< 0.0001	0.97 (0.80–1.17)	0.7637

*SBP* systolic blood pressure; *DBP* diastolic blood pressure; *HDL-C* high density lipoprotein cholesterol; *FPG* fasting plasma glucose; *HbA1c* glycated hemoglobin; *HOMA-IR* homeostasis model assessment of insulin resistance; *BMI* body mass index; *MUHS* metabolic unhealthy and hepatic steatosis; *OR* odds ratio; *CI* confidence interval

^†^Logistic regression models with generalized estimating equations were adjusted for age, sex, current smoking and drinking status (yes/no), education (≥ 12 years or not), log-transformed physical activity, waist circumference, SBP, DBP, triglycerides, HDL-C, FPG, 2 h-PG, HbA1c, HOMA-IR and BMI. All covariates with the exception for sex and education were repeated measured both at baseline and follow-up and modelled as time-varying variables

## Discussion

On the basis of metabolic abnormalities proposed by MAFLD criteria, this prospective cohort study evaluated the overall metabolic status among participants with/without fatty liver disease. Increased risk of subclinical atherosclerosis was observed in varying combinations of fatty liver status and metabolic abnormalities, defining as the presence of type 2 diabetes and/or ≥ 2 metabolic risk factors, except MHHS group. During the 4.3-year follow-up period, mostly proportions of participants either maintained MU status or progressed from MH to MU status, which impelled the development of subclinical atherosclerosis. In contrast to the metabolic progression, MU regression exhibited a mitigative effect on the composite risk, especially among participants with fatty liver disease. MUHS participants regressing to MH status tended to have a more prominent improvement on waist circumference, SBP, triglycerides, HDL-C, HbA1c, fasting and postprandial plasma glucose.

Since changing term from NAFLD to MAFLD, fatty liver disease associated with metabolic abnormalities is of growingly public health concern. Previous evidence has been explicit that NAFLD or the new term MAFLD was an independent risk factor for both clinical cardiovascular events [23–25] and subclinical atherosclerotic lesion [26, 27]. Results in the prior half of the current study were generally in line with previous cohort findings, supporting the notion that fatty liver disease combining with metabolic abnormalities synergistically contributed to the increased risk of subclinical atherosclerosis, reflected by artery stiffness (elevated baPWV and PP) and endothelial dysfunction (albuminuria). MHHS group, representing the phenotype who developed simple hepatic steatosis ahead of metabolic dysfunction, did not show significant association with the subclinical atherosclerotic risk in our cohort. The results were consistent with a Korean cross-sectional study comparing hepatic fibrosis and cardiovascular risk among the MH-MAFLD with healthy control [10]. Whether the neutralized effect or the limitation of sample size matters, more data are needed in the future.

On the other hand, metabolic status transition is complex and dynamically changing over time. Nevertheless, long-term data regarding the metabolic status transition in fatty liver population are still limited. Previous data were mainly referred to metabolic syndrome among participants with obesity. Corresponding transition rate of MH progression was about 40% to 50% over 8–20 follow-up years in different populations [28–31]. Additionally, the MH individuals had the potential to progress to MU status over time across all BMI categories. The transition accompanying with a greater degree of adiposity sequenced a higher cardiovascular risk [32]. Even though the follow-up duration of our cohort was relatively short, we observed 17.6% of metabolic transition, in which participants characterized with older age, lower educational attainment and higher levels of cardiometabolic parameters, even in the normal-high range, were prone to progress to MU status. Additionally, participants with fatty liver were more predisposed to be stuck in the MU status, which contributed to a more evident risk of developing subclinical atherosclerosis. Pooling all the evidence highlighted the necessity of close monitor and prompt intervention to impede the metabolic progression, especially among those who were concomitant with general or visceral fat deposition.

In addition to the metabolic progression, regressing from MU to MH status benefits not only to the systematic metabolic profile but also to the related complications. In a Korean nationwide cohort study, regression of MU status among participants with normal weight and obesity was significantly related to decreased risk of incident cardiovascular events and all-cause mortality [33]. Similarly, the evident benefit on the composite risk was discovered in the current study when regressing to MH status, especially among participants with fatty liver disease. This beneficial effect was not influenced by metabolic medications and more likely to be attributed to lifestyle modification, indicating its cornerstone position in the treatment of fatty liver disease. Furthermore, findings from our previous research manifested that baseline MAFLD participants with low probability of fibrosis regressing to non-MAFLD at follow-up decreased the risk of elevated baPWV by 43.1% [27]. Findings may need to be verified in a larger-scale and longer-term cohort, nevertheless, our study provided the evidence to some extent that recommendations for the primary cardiometabolic risk prevention should not overlook the importance of maintaining MH status regardless of fatty liver status.

The precise determinants responsible for MH progression have not been fully understood. A multitude of factors, including genetics, age, waist circumference, BMI, lipids, glycemic parameters, poor dietary quality, physical inactivity and gut microbiota, interact in a complex and dynamic manner to influence individuals’ MH status [34–36]. Moreover, accumulating visceral fat was found to be associated with progressing to MU phenotype while decreasing visceral fat mass was associated with MU regression [37]. A recent prospective cohort study also manifested that the presence of NAFLD facilitated both MH-obesity progression and 10-year cardiovascular disease risk [38]. Our study further investigated that participants with MU regression were more likely to be associated with improvement of waist circumference, blood pressure, lipids and glucose, which implicated the priority to pay constant attention to among fatty liver population. Findings from a nationwide cohort study also demonstrated that if NAFLD participants with prediabetes or diabetes could achieve ≥ 2 of metabolic goals towards glycemia, blood pressure or lipids, risk of cardiovascular and chronic kidney disease would be mitigated [39]. Pathophysiology of increased visceral fat mass in relation to atherosclerosis and cardiometabolic diseases can be mainly explained by insulin resistance, subclinical inflammation, dysregulated adipokine secretion and increased release of fatty acids into the circulation, implying potential targets of therapy for fatty liver disease and metabolic comorbidities [40, 41]. Experimental studies have shown that diets enriched with omega-3 polyunsaturated fatty acids increase insulin sensitivity, reduce intrahepatic triglyceride content and ameliorate steatohepatitis [42].Moreover, the Mediterranean diet plays a beneficial role in metabolic profile and has been shown to reduce the risk of cardiovascular disease and diabetes, two outcomes highly relevant in NAFLD patients [43, 44].

A close relationship between glycemic control and fatty liver disease was found in this study. Given the most evident significance was presented in the group of fatty liver combining with diabetes and glucose parameters were all involved in the MU regression, glycemic control would be a more prominent target towards related prognosis among fatty liver population.

To our knowledge, this was the first prospective study with the specific aim to evaluate the associations of overall metabolic status among participants with/without fatty liver disease, and further metabolic transition with the risk of incident subclinical atherosclerosis, under the context of metabolic abnormalities proposed by MAFLD definition. The strengths of our study included the prospective design and the investigation of effects towards varying metabolic transition on incident subclinical atherosclerosis risk. Factors in relation to the metabolic regression among fatty liver population were further explored using the repeated measures analysis. Our findings provided certain evidence towards the critical role of metabolic abnormalities in the course of fatty liver disease, as highlighted in MAFLD definition. Additionally, MHHS participants were not definitely found to be associated with subclinical atherosclerosis risk during the follow-up period, while baseline characteristics with the youngest age, highest level of education, as well as drinking and smoking habits, indicating the optimal matching to long-term lifestyle intervention. For MU individuals, accurate assessment, targeted treatment and intensive follow-up were necessary to impede the progressive course, especially among patients who exposed to continuous hepatic steatosis.

Several limitations still merited to mention. First, the inflammatory indicator of high-sensitive C-reaction protein was not measured or included in the criteria of MU status. Second, the current study may not be sufficiently powered to assess the associations of MHHS and further MU regression with subclinical atherosclerotic risk, due to the relatively small sample and short follow-up duration. Third, causal explanations were not accessible with a single follow-up visit, in that the metabolic transition and subclinical outcomes developed in parallel. Fourth, confounders were adjusted based on knowledge of their associations with MAFLD and subclinical atherosclerosis. Lifestyle modification, such as smoking, alcohol drinking, physical activity and body weight management, remains the cornerstone for MAFLD treatment [45]. Additionally, improving cardiovascular health has been a target for prevention of MAFLD as well as subclinical atherosclerosis and cardiovascular disease [15, 46]. Despite of the discreet adjustments for potential confounders, the possibility of residual confounding factors due to uncollected variables such as dietary information cannot be excluded. Therefore, those significant implications warranted to be consolidated in a cohort with wider-generalizability.

## Conclusion

In summary, the current study built upon the emphasis to better evaluate overall metabolic status and drew a synergistic effect of fatty liver disease along with metabolic abnormalities on the increased risk of subclinical atherosclerosis. During up to 5-year follow-up, participants who were caught in the metabolic exacerbation had an increased risk of subclinical atherosclerosis, while fatty liver participants achieving MU regression were more intended to mitigate the risk. Undoubtedly, MH warrants to be maintained and defended via a comprehensive management, especially the improvement of waist circumference, blood pressure, glucose and lipids among fatty liver population which were highlighted in our study. In the era of precision medicine, multifaceted risk stratification will conduce to optimize the efficacy and cost-effectiveness of diagnosis and targeted intervention.

## Supplementary Information

**Additional file 1**.** Table S1**. Risk of incident subclinical atherosclerosis (composite/separate) according to MH/MU and fatty liver status at baseline excluding FIB-4 > 2.67, other liver diseases and excessive alcohol consumption.** Table S2**. Baseline characteristics according to the metabolic transition.** Table S3**. Transition of MH status and the risk of composite subclinical atherosclerosis without glucose-, blood pressure- or lipid-lowering pharmacological treatment.** Table S4**. Transition of MH status and the risk of composite subclinical atherosclerosis excluding FIB-4 > 2.67, other liver diseases and excessive alcohol consumption.** Table S5**. Effect of metabolic status improvement on composite risk of subclinical atherosclerosis among MU population without glucose-, blood pressure- or lipid-lowering pharmacological treatment.** Table S6**. Effect of metabolic status improvement on composite risk of subclinical atherosclerosis among MU population excluding FIB-4 > 2.67, other liver diseases and excessive alcohol consumption.** Table S7**. The effect of metabolic status improvement on separate subclinical atherosclerosis risk among MU population.** Table S8**. The effect of metabolic status improvement on separate subclinical atherosclerosis risk among MU population without glucose-, blood pressure- or lipid-lowering pharmacological treatment.** Table S9**. Changes of metabolic risk factors between stable MU and MU to MH groups among baseline MUHS participants.** Table S10**. Baseline characteristics of participants included and those lost to follow-up.

## Data Availability

The datasets generated and/or analyzed are available from the corresponding author on reasonable request.

## Abbreviations

2 h-PG: 2H-postprandial glucose
baPWV: Brachial-ankle pulse wave velocity
BMI: Body mass index
CI: Confidence interval
DBP: Diastolic blood pressure
FIB-4: Fibrosis-4 score
FPG: Fasting plasma glucose
HbA1c: Glycated hemoglobin
HDL-C: High-density lipoprotein cholesterol
HOMA-IR: Homeostasis model assessment of insulin resistance
LDL-C: Low-density lipoprotein cholesterol
MAFLD: Metabolic dysfunction-associated fatty liver disease
MET-min/wk: Metabolic equivalent minute per week
MH: Metabolic healthy
MHHS: Metabolic healthy and hepatic steatosis
MHNHS: Metabolic healthy and no hepatic steatosis
MU: Metabolic unhealthy
MUHS: Metabolic unhealthy and hepatic steatosis
MUNHS: Metabolic unhealthy and no hepatic steatosis
NAFLD: Non-alcoholic fatty liver disease
OR: Odds ratio
PP: Pressure pulse
SBP: Systolic blood pressure
UACR: Urinary albumin-to-creatinine ratio
