# Supplementary material for: Risk of subclinical atherosclerosis across metabolic transition in individuals with or without fatty liver disease: a prospective cohort study
Source: Nutr Metab (Lond). 2023 Mar 10;20:15. doi: 10.1186/s12986-023-00734-3 (PMC10007748; doi:10.1186/s12986-023-00734-3)
Supplement: Supplementary file 1 — Additional file 1. Table S1. Risk of incident subclinical atherosclerosis (composite/separate) according to MH/MU and fatty liver status at baseline excluding FIB-4 > 2.67, other liver diseases and excessive alcohol consumption. Table S2. Baseline characteristics according to the metabolic transition. Table S3. Transition of MH status and the risk of composite subclinical atherosclerosis without glucose-, blood pressure- or lipid-lowering pharmacological treatment. Table S4. Transition of MH status and the risk of composite subclinical atherosclerosis excluding FIB-4 > 2.67, other liver diseases and excessive alcohol consumption. Table S5. Effect of metabolic status improvement on composite risk of subclinical atherosclerosis among MU population without glucose-, blood pressure- or lipid-lowering pharmacological treatment. Table S6. Effect of metabolic status improvement on composite risk of subclinical atherosclerosis among MU population excluding FIB-4 > 2.67, other liver diseases and excessive alcohol consumption. Table S7. The effect of metabolic status improvement on separate subclinical atherosclerosis risk among MU population. Table S8. The effect of metabolic status improvement on separate subclinical atherosclerosis risk among MU population without glucose-, blood pressure- or lipid-lowering pharmacological treatment. Table S9. Changes of metabolic risk factors between stable MU and MU to MH groups among baseline MUHS participants. Table S10. Baseline characteristics of participants included and those lost to follow-up. [file 12986_2023_734_MOESM1_ESM.docx]

**Table of contents**

| **Table S1** Risk of incident subclinical atherosclerosis (composite/separate) according to MH/MU and fatty liver status at baseline excluding FIB-4 > 2.67, other liver diseases and excessive alcohol consumption | Page 2 |
| --- | --- |
| **Table S2.** Baseline characteristics according to the metabolic transition. | Page 3 |
| **Table S3** Transition of MH status and the risk of composite subclinical atherosclerosis without glucose-, blood pressure- or lipid-lowering pharmacological treatment | Page 4 |
| **Table S4** Transition of MH status and the risk of composite subclinical atherosclerosis excluding FIB-4 > 2.67, other liver diseases and excessive alcohol consumption | Page 5 |
| **Table S5** Effect of metabolic status improvement on composite risk of subclinical atherosclerosis among MU population without glucose-, blood pressure- or lipid-lowering pharmacological treatment | Page 6 |
| **Table S6** Effect of metabolic status improvement on composite risk of subclinical atherosclerosis among MU population excluding FIB-4 > 2.67, other liver diseases and excessive alcohol consumption | Page 7 |
| **Table S7** The effect of metabolic status improvement on separate subclinical atherosclerosis risk among MU population | Page 8 |
| **Table S8** The effect of metabolic status improvement on separate subclinical atherosclerosis risk among MU population without glucose-, blood pressure- or lipid-lowering pharmacological treatment | Page 9 |
| **Table S9** Changes of metabolic risk factors between stable MU and MU to MH groups among baseline MUHS participants | Page 10 |
| **Table S10** Baseline characteristics of participants included and those lost to follow-up | Page 11 |

**Table S1 Risk of incident subclinical atherosclerosis (composite/separate) according to MH/MU and fatty liver status at baseline excluding FIB-4 > 2.67, other liver diseases and excessive alcohol consumption**

|  | **N** | **Case, n (%)** | **OR (95% CI)*** | ***P* value** |
| --- | --- | --- | --- | --- |
| **Composite Subclinical atherosclerosis (725/3141, 23.1%)** | | | | |
| MHNHS | 923 | 111 (12.0) | 1.00 | - |
| MUNHS | 1350 | 359 (26.6) | 1.76 (1.33-2.33) | < 0.0001 |
| MHHS | 45 | 8 (17.8) | 1.47 (0.57-3.76) | 0.4258 |
| MUHS | 823 | 247 (30.0) | 2.52 (1.80-3.53) | < 0.0001 |
| **Elevated baPWV (637/3891, 16.4%)** | | | | |
| MHNHS | 1030 | 82 (8.0) | 1.00 | - |
| MUNHS | 1733 | 328 (18.9) | 1.91 (1.38-2.64) | < 0.0001 |
| MHHS | 47 | 5 (10.6) | 1.81 (0.59-5.55) | 0.2973 |
| MUHS | 1081 | 222 (20.5) | 2.62 (1.80-3.83) | < 0.0001 |
| **Elevated PP (512/3890, 13.2%)** | | | | |
| MHNHS | 1040 | 52 (5.0) | 1.00 | - |
| MUNHS | 1710 | 278 (16.3) | 2.88 (1.97-4.21) | < 0.0001 |
| MHHS | 48 | 3 (6.3) | 2.14 (0.61-7.52) | 0.2376 |
| MUHS | 1092 | 179 (16.4) | 3.24 (2.11-4.99) | < 0.0001 |
| **Albuminuria (361/4715, 7.7%)** | | | | |
| MHNHS | 1068 | 51 (4.8) | 1.00 | - |
| MUNHS | 2163 | 155 (7.2) | 1.01 (0.68-1.50) | 0.9499 |
| MHHS | 51 | 3 (5.9) | 1.09 (0.25-4.74) | 0.9138 |
| MUHS | 1433 | 152 (10.6) | 1.83 (1.18-2.82) | 0.0065 |

*OR (95% CI) was further adjusted for current smoking and drinking status (yes/no), education (≥ 12 years or not), log-transformed physical activity, baseline BMI and BMI change.

FIB-4, Fibrosis-4 score; MHNHS, metabolic healthy and no hepatic steatosis; MUNHS, metabolic unhealthy and no hepatic steatosis; MHHS, metabolic healthy and hepatic steatosis; MUHS, metabolic unhealthy and hepatic steatosis; baPWV, brachial–ankle pulse wave velocity; PP, pulse pressure; OR, odds ratio; BMI, body mass index.

**Table S2. Baseline characteristics according to the metabolic transition**

|  | Stable MH  (n=713) | MH to MU  (n=732) | Stable MU  (n=4444) | MU to MH  (n=371) | *P* for trend |
| --- | --- | --- | --- | --- | --- |
| Age, year | 53.5±8.3 | 55.8±8.2 | 58.8±8.5 | 56.3±8.5 | < 0.0001 |
| Male, n (%) | 273 (38.3) | 308 (42.1) | 1515 (34.1) | 168 (45.3) | < 0.0001 |
| High school and above, n (%) | 216 (30.3) | 156 (21.3) | 796 (17.9) | 89 (24.0) | < 0.0001 |
| Current drinking, n (%) | 161 (22.6) | 171 (23.4) | 787 (17.7) | 95 (25.6) | 0.0266 |
| Current smoking, n (%) | 197 (27.6) | 196 (26.8) | 902 (20.3) | 116 (31.3) | 0.0037 |
| Vigorous activity ≥ 75 min/week or moderate-vigorous ≥ 150 min/week, n (%) | 118 (16.6) | 96 (13.1) | 748 (16.8) | 52 (14.0) | 0.6618 |
| Body mass index (kg/m^2^) | 22.5±2.2 | 23.4±2.3 | 26.1±3.2 | 23.9±2.6 | < 0.0001 |
| Waist circumference (cm) | 74.6±6.5 | 77.1±6.6 | 85.2±8.3 | 79.9±7.4 | < 0.0001 |
| SBP (mmHg) | 123.7±15.4 | 131.8±17.7 | 146.2±18.7 | 135.5±16.5 | < 0.0001 |
| DBP (mmHg) | 75.8±8.6 | 79.6±9.8 | 85.0±10.0 | 81.8±8.4 | < 0.0001 |
| Fasting plasma glucose (mg/dL) | 86.3±7.7 | 89.4±8.1 | 104.9±30.3 | 89.3±9.2 | < 0.0001 |
| Postprandial plasma glucose (mg/dL) | 101.4±23.7 | 106.8±24.2 | 165.7±84.8 | 113.8±28.0 | < 0.0001 |
| HbA1c (%) | 5.4±0.3 | 5.4±0.3 | 6.0±1.0 | 5.6±0.4 | < 0.0001 |
| HOMA-IR | 1.0 (0.7-1.3) | 1.1 (0.7-1.5) | 1.9 (1.3-2.9) | 1.3 (0.9-1.9) | < 0.0001 |
| Triglycerides (mg/dL) | 80.5  (64.6-104.4) | 95.1  (73.5-118.6) | 142.5  (101.8-197.3) | 108.0  (77.0-154.9) | < 0.0001 |
| LDL-C (mg/dL) | 110.1±27.4 | 118.6±29.4 | 127.4±34.6 | 121.0±30.0 | < 0.0001 |
| HDL-C (mg/dL) | 59.1±11.0 | 56.5±11.0 | 48.8±11.5 | 53.0±14.4 | < 0.0001 |
| Total cholesterol (mg/dL) | 193.9±32.3 | 200.2±34.1 | 210.9±40.5 | 201.1±34.9 | < 0.0001 |

Values are means ± standard deviations, medians (interquartile ranges) or numbers (proportions).

Abbreviations: SBP, systolic blood pressure; DBP, diastolic blood pressure; HbA1c, glycated hemoglobin; HOMA-IR, homeostasis model assessment of insulin resistance; LDL-C, low density lipoprotein cholesterol; HDL-C, high density lipoprotein cholesterol; MH, metabolic health; MU, metabolic unhealth.

**Table S3 Transition of MH status and the risk of composite subclinical atherosclerosis without glucose-, blood pressure- or lipid-lowering pharmacological treatment**

|  | **No. cases/participants, (%)** | **OR (95% CI)*** | ***P* value** |
| --- | --- | --- | --- |
| **No fatty liver at baseline** | | | |
| Stable MH | 44/563 (7.8) | 1.00 |  |
| MH to MU | 71/409 (17.4) | 2.375 (1.474-3.828) | 0.0004 |
| Stable MU | 200/749 (26.7) | 3.262 (2.112-5.038) | < 0.0001 |
| MU to MH | 22/217 (10.1) | 0.953 (0.500-1.815) | 0.8836 |
| **Fatty liver at baseline** | | | |
| Stable MH | 0/13 (0) | <0.001 (<0.001->999.999) | 0.9803 |
| MH to MU | 6/32 (18.8) | 2.913 (0.985-8.616) | 0.0533 |
| Stable MU | 104/389 (26.7) | 3.979 (2.378-6.659) | < 0.0001 |
| MU to MH | 2/34 (5.9) | 0.466 (0.060-3.633) | 0.4660 |

*OR (95% CI) was adjusted for age, sex, follow-up interval, current smoking and drinking status (yes/no), education (≥ 12 years or not), log (physical activity), baseline BMI and BMI change.

MH, meatabolic health; MU, metabolic unhealth; OR, odds ratio; CI, confidence interval; BMI, body mass index.

**Table S4 Transition of MH status and the risk of composite subclinical atherosclerosis excluding FIB-4 > 2.67, other liver diseases and excessive alcohol consumption**

|  | **No. cases/participants, (%)** | **OR (95% CI)*** | ***P* value** |
| --- | --- | --- | --- |
| **No fatty liver at baseline** | | | |
| Stable MH | 39/502 (7.8) | 1.00 | - |
| MH to MU | 72/421 (17.1) | 2.33 (1.45-3.75) | 0.0005 |
| Stable MU | 335/1160 (28.9) | 3.25 (2.14-4.92) | < 0.0001 |
| MU to MH | 24/190 (12.6) | 1.09 (0.57-2.11) | 0.7873 |
| **Fatty liver at baseline** | | | |
| Stable MH | 0/10 (0) | <0.001 (<0.001->999.999) | 0.9736 |
| MH to MU | 8/35 (22.9) | 3.05 (1.11-8.40) | 0.0311 |
| Stable MU | 244/789 (30.9) | 4.51 (2.86-7.12) | < 0.0001 |
| MU to MH | 3/34 (8.8) | 0.78 (0.17-3.50) | 0.7403 |

*OR (95% CI) was adjusted for age, sex, follow-up interval, current smoking and drinking status (yes/no), education (≥ 12 years or not), log (physical activity), baseline BMI and BMI change.

FIB-4, Fibrosis-4 score; MH, meatabolic health; MU, metabolic unhealth; OR, odds ratio; CI, confidence interval; BMI, body mass index.

**Table S5 Effect of metabolic status improvement on composite risk of subclinical atherosclerosis among MU population without glucose-, blood pressure- or lipid-lowering pharmacological treatment**

|  | **No. participants** | **Case, n (%)** | **OR (95% CI)*** | ***P* value** |
| --- | --- | --- | --- | --- |
| Stable MU | 1138 | 304 (26.7) | 1.00 |  |
| MU to MH | 251 | 24 (9.56) | 0.27 (0.16-0.47) | < 0.0001 |
| **Fatty liver** |  |  |  |  |
| Stable MU | 389 | 104 (26.74) | 1.00 |  |
| MU to MH | 34 | 2 (5.88) | 0.12 (0.02-0.96) | 0.0451 |
| **No fatty liver** |  |  |  |  |
| Stable MU | 749 | 200 (26.70) | 1.00 |  |
| MU to MH | 217 | 22 (10.14) | 0.29 (0.16-0.52) | < 0.0001 |

*OR (95% CI) was adjusted for age, sex, follow-up interval, current smoking and drinking status (yes/no), education (≥ 12 years or not), log-transformed physical activity, baseline BMI and BMI change.

MH, meatabolic healthy; MU, metabolic unhealthy; OR, odds ratio; CI, confidence interval; BMI, body mass index.

**Table S6 Effect of metabolic status improvement on composite risk of subclinical atherosclerosis among MU population excluding FIB-4 > 2.67, other liver diseases and excessive alcohol consumption**

|  | **No. participants** | **Case, n (%)** | **OR (95% CI)*** | ***P* value** |
| --- | --- | --- | --- | --- |
| Stable MU | 1949 | 579 (29.7) | 1.00 | - |
| MU to MH | 224 | 27 (12.1) | 0.30 (0.18-0.50) | < 0.0001 |
| **Fatty liver** |  |  |  |  |
| Stable MU | 789 | 244 (30.9) | 1.00 | - |
| MU to MH | 34 | 3 (8.2) | 0.18 (0.04-0.77) | 0.0213 |
| **No fatty liver** |  |  |  |  |
| Stable MU | 1160 | 335 (28.9) | 1.00 | - |
| MU to MH | 190 | 24 (12.6) | 0.33 (0.19-0.59) | 0.0001 |

*OR (95% CI) was adjusted for age, sex, follow-up interval, current smoking and drinking status (yes/no), education (≥ 12 years or not), log-transformed physical activity, baseline BMI and BMI change.

FIB-4, Fibrosis-4 score; MH, meatabolic healthy; MU, metabolic unhealthy; OR, odds ratio; CI, confidence interval; BMI, body mass index.

**Table S7 The effect of metabolic status improvement on separate subclinical atherosclerosis risk among MU population**

|  | **No. participants** | **Case, n (%)** | **OR (95% CI)*** | ***P* value** |
| --- | --- | --- | --- | --- |
| **Elevated baPWV** | 3379 | 703 (20.8) |  |  |
| Stable MU | 3046 | 673 (22.1) | 1.00 | - |
| MU to MH | 333 | 30 (9.0) | 0.31 (0.19-0.51) | < 0.0001 |
| Fatty liver | 1272 | 273 (21.5) |  |  |
| Stable MU | 1227 | 271 (22.1) | 1.00 | - |
| MU to MH | 45 | 2 (4.4) | 0.24 (0.05-1.01) | 0.0517 |
| No fatty liver | 2107 | 430 (20.4) |  |  |
| Stable MU | 1819 | 402 (22.1) | 1.00 |  |
| MU to MH | 288 | 28 (9.7) | 0.33 (0.19-0.55) | < 0.0001 |
| **Elevated PP** | 3363 | 552 (16.4) |  |  |
| Stable MU | 3042 | 531 (17.5) | 1.00 |  |
| MU to MH | 321 | 21 (6.5) | 0.36 (0.21-0.61) | 0.0001 |
| Fatty liver | 1285 | 214 (16.7) |  |  |
| Stable MU | 1241 | 213 (17.2) | 1.00 |  |
| MU to MH | 44 | 1 (2.3) | 0.16 (0.02-1.22) | 0.0775 |
| No fatty liver | 2078 | 338 (16.3) |  |  |
| Stable MU | 1801 | 318 (17.7) | 1.00 |  |
| MU to MH | 277 | 20 (7.2) | 0.42 (0.25-0.74) | 0.0023 |
| **Albuminuria** | 4388 | 403 (9.2) |  |  |
| Stable MU | 4037 | 394 (9.8) | 1.00 |  |
| MU to MH | 351 | 9 (2.6) | 0.29 (0.13-0.66) | 0.0034 |
| Fatty liver | 1704 | 192 (11.3) |  |  |
| Stable MU | 1659 | 190 (11.5) | 1.00 |  |
| MU to MH | 45 | 2 (4.4) | 0.32 (0.04-2.40) | 0.2679 |
| No fatty liver | 2684 | 211 (7.9) |  |  |
| Stable MU | 2378 | 204 (8.6) | 1.00 |  |
| MU to MH | 306 | 7 (2.3) | 0.31 (0.12-0.78) | 0.0128 |

*OR (95% CI) was adjusted for age, sex, follow-up interval, current smoking and drinking status (yes/no), education (≥ 12 years or not), log-transformed physical activity, baseline BMI and BMI change.

MH, meatabolic healthy; MU, metabolic unhealthy; OR, odds ratio; CI, confidence interval; baPWV, brachial–ankle pulse wave velocity; PP, pulse pressure; BMI, body mass index.

**Table S8 The effect of metabolic status improvement on separate subclinical atherosclerosis risk among MU population without glucose-, blood pressure- or lipid-lowering pharmacological treatment**

|  | **No. participants** | **Case, n (%)** | **OR (95% CI)*** | ***P* value** |
| --- | --- | --- | --- | --- |
| **Elevated baPWV** | 1617 | 235 (14.5) |  |  |
| Stable MU | 1335 | 217 (16.3) | 1.00 |  |
| MU to MH | 282 | 18 (6.4) | 0.28 (0.15-0.52) | < 0.0001 |
| Fatty liver | 487 | 70 (14.4) |  |  |
| Stable MU | 451 | 69 (15.3) | 1.00 |  |
| MU to MH | 36 | 1 (2.8) | 0.29 (0.04-2.31) | 0.2426 |
| No fatty liver | 1130 | 165 (14.6) |  |  |
| Stable MU | 884 | 148 (16.7) | 1.00 |  |
| MU to MH | 246 | 17 (6.9) | 0.27 (0.14-0.53) | 0.0001 |
| **Elevated PP** | 1633 | 218 (13.4) |  |  |
| Stable MU | 1357 | 205 (15.1) | 1.00 |  |
| MU to MH | 276 | 13 (4.7) | 0.32 (0.17-0.63) | 0.0008 |
| Fatty liver | 496 | 65 (13.1) |  |  |
| Stable MU | 461 | 64 (13.9) | 1.00 |  |
| MU to MH | 35 | 1 (2.9) | 0.24 (0.03-1.89) | 0.1743 |
| No fatty liver | 1137 | 153 (13.5) |  |  |
| Stable MU | 896 | 141 (15.7) | 1.00 |  |
| MU to MH | 241 | 12 (5.0) | 0.35 (0.17-0.70) | 0.0030 |
| **Albuminuria** | 1826 | 118 (6.5) |  |  |
| Stable MU | 1535 | 110 (7.2) | 1.00 |  |
| MU to MH | 291 | 8 (2.8) | 0.43 (0.17-1.10) | 0.0788 |
| Fatty liver | 551 | 47 (8.5) |  |  |
| Stable MU | 513 | 46 (9.0) | 1.00 |  |
| MU to MH | 38 | 1 (2.6) | < 0.001 (<0.001->999.999) | 0.9713 |
| No fatty liver | 1275 | 71 (5.6) |  |  |
| Stable MU | 1022 | 64 (6.3) | 1.00 |  |
| MU to MH | 253 | 7 (2.8) | 0.50 (0.19-1.32) | 0.1598 |

*OR (95% CI) was adjusted for age, sex, follow-up interval, current smoking and drinking status (yes/no), education (≥ 12 years or not), log-transformed physical activity, baseline BMI and BMI change.

MH, meatabolic healthy; MU, metabolic unhealthy; OR, odds ratio; CI, confidence interval; baPWV, brachial–ankle pulse wave velocity; PP, pulse pressure; BMI, body mass index.

**Table S9 Changes of metabolic risk factors between stable MU and MU to MH groups among baseline MUHS participants**

|  | **Stable MU** | **MU to MH** | ***P* value** |
| --- | --- | --- | --- |
| No. of participants, n (%) | 1848 (97.5) | 48 (2.5) | - |
| *Δ* Waist circumference, cm | 0.0±7.2 | -3.8±4.6 | 0.0003 |
| *Δ* SBP, mmHg | -8.3±17.0 | -9.8±13.0 | 0.5502 |
| *Δ* DBP, mmHg | -8.5±9.5 | -9.9±6.9 | 0.3163 |
| *Δ* Triglycerides, mg/dL | 4.4 (-41.6, 52.5) | -22.6 (-45.6, -6.2) | 0.0160 |
| *Δ* HDL-C, mg/dL | 3.2±84.2 | 3.6±7.5 | 0.9747 |
| *Δ* FPG, mg/dL | 10.8±31.0 | 6.2±9.4 | 0.3041 |
| *Δ* 2h-PG, mg/dL | 9.1±75.5 | 0.4±31.6 | 0.4302 |
| *Δ* HbA1c, % | -0.1±1.0 | -0.2±0.3 | 0.6262 |
| *Δ* HOMA-IR | 0.3 (-0.6, 1.1) | -0.3 (-0.9, 0.2) | 0.0021 |
| *Δ* BMI, kg/m^2^ | 0.6±1.8 | 1.3±1.5 | 0.0024 |

Changes of metabolic risk parameters are presented as means ± standard deviations, medians (interquartile ranges) or numbers (proportions).

Negative numbers indicated that the levels of metabolic risk parameters at follow-up were lower than the baseline. *P* values were calculated from *t* test for comparison between the stable MU and MU to MH groups.

MUHS, metabolic unhealthy and hepatic steatosis; MH, metabolic healthy; SBP, systolic blood pressure; DBP, diastolic blood pressure; HDL-C, high density lipoprotein cholesterol; FPG, fasting plasma glucose; HbA1c, glycated hemoglobin; HOMA-IR, homeostasis model assessment of insulin resistance; BMI, body mass index.

**Table S10 Baseline characteristics of participants included and those lost to follow-up**

| **Characteristics** | **Participants included in the study**  **(n=6260)** | **Participants lost to follow-up**  **(n=3541)** |
| --- | --- | --- |
| Age (years) | 57.7±8.6 | 58.6±10.7 |
| Male, n (%) | 2264 (36.2) | 1409 (39.8) |
| High school and above, n (%) | 1257 (20.1) | 900 (25.4) |
| Current drinking, n (%) | 1214 (19.4) | 754 (21.3) |
| Current smoking, n (%) | 1411 (22.5) | 860 (24.3) |
| Vigorous activity ≥ 75 min/week or moderate-vigorous ≥ 150 min/week, n (%) | 1014 (16.2) | 547 (15.5) |
| Hypertension, n (%) | 3696 (59.0) | 2038 (57.6) |
| Anti-hypertensive medications, n (%) | 4471 (71.4) | 2551 (72.0) |
| Type 2 diabetes, n (%) | 1207 (19.3) | 636 (18.0) |
| Anti-diabetic medications or insulin, n (%) | 458 (7.3) | 235 (6.6) |
| Dyslipidemia, n (%) | 2645 (42.3) | 1456 (41.1) |
| Lipid lowering medications, n (%) | 14 (0.2) | 11 (0.3) |
| Body mass index (kg/m^2^) | 25.3±3.3 | 25.0±3.3 |
| Waist circumference (cm) | 82.7±8.9 | 82.8±9.2 |
| SBP (mmHg) | 141.3±19.8 | 139.9±20.3 |
| DBP (mmHg) | 83.1±10.3 | 82.3±10.3 |
| PP (mmHg) | 58.2±15.7 | 57.6±16.6 |
| Fasting plasma glucose (mg/dL) | 100.0±27.0 | 99.1±25.9 |
| Postprandial plasma glucose (mg/dL) | 148.4±77.8 | 147.0±74.9 |
| HbA1c (%) | 5.8±0.9 | 5.8±0.9 |
| HOMA-IR | 1.6 (1.1-2.5) | 1.6 (1.1-2.5) |
| Triglycerides (mg/dL) | 123.0 (88.5-174.3) | 121.2 (85.0-172.6) |
| LDL-C (mg/dL) | 124.0±33.5 | 122.9±33.2 |
| HDL-C (mg/dL) | 51.1±12.2 | 51.2±12.0 |
| Total cholesterol (mg/dL) | 207.1±39.1 | 205.5±39.5 |
| ALT (IU) | 18.2 (14.0-25.4) | 17.7 (13.4-25.1) |
| AST (IU) | 21.5 (18.4-25.5) | 21.3 (18.1-25.3) |
| GGT (IU) | 21.0 (15.0-34.0) | 22.0 (15.0-34.0) |
| BaPWV (cm/s) | 1598.5±352.5 | 1619.3±386.6 |
| UACR (mg/g) | 4.9 (2.8-9.0) | 4.9 (2.8-9.1) |

Values are means ± standard deviations, medians (interquartile ranges) or numbers (proportions).

Abbreviations: SBP, systolic blood pressure; DBP, diastolic blood pressure; PP, pulse pressure; HbA1c, glycated hemoglobin; HOMA-IR, homeostasis model assessment of insulin resistance; LDL-C, low density lipoprotein cholesterol; HDL-C, high density lipoprotein cholesterol; ALT, alanine aminotransferase; AST, aspartate aminotransferase; GGT, gamma-glutamyl transferase; baPWV, brachial–ankle pulse wave velocity; UACR, urinary albumin-to-creatinine ratio.
